# The impact of parametrized convection on cloud feedback

**DOI:** 10.1098/rsta.2014.0414

**Published:** 2015-11-13

**Authors:** Mark J. Webb, Adrian P. Lock, Christopher S. Bretherton, Sandrine Bony, Jason N. S. Cole, Abderrahmane Idelkadi, Sarah M. Kang, Tsuyoshi Koshiro, Hideaki Kawai, Tomoo Ogura, Romain Roehrig, Yechul Shin, Thorsten Mauritsen, Steven C. Sherwood, Jessica Vial, Masahiro Watanabe, Matthew D. Woelfle, Ming Zhao

**Affiliations:** 1 Met Office Hadley Centre (MOHC), Exeter, UK; 2 University of Washington (UW), Seattle, WA, USA; 3 Laboratoire de Météorologie Dynamique/Institute Pierre Simon Laplace (IPSL), Paris, France; 4 Canadian Centre for Climate Modelling and Analysis (CCCma), Victoria, British Columbia, Canada; 5 Ulsan National Institute of Science and Technology (UNIST), Ulsan, Republic of Korea; 6 Meteorological Research Institute (MRI), Tsukuba, Ibaraki Prefecture, Japan; 7 National Institute for Environmental Studies (NIES), Tsukuba, Ibaraki Prefecture, Japan; 8 Centre National de Recherches Météorologiques (CNRM), Toulouse, France; 9 Max Planck Institute for Meteorology (MPI-M), Hamburg, Germany; 10 University of New South Wales (UNSW), Sydney, New South Wales, Australia; 11 Atmosphere and Ocean Research Institute (AORI), Chiba, Japan; 12 Geophysical Fluid Dynamics Laboratory (GFDL), Princeton, NJ, USA

**Keywords:** cloud, climate, feedback, convection, parametrization

## Abstract

We investigate the sensitivity of cloud feedbacks to the use of convective parametrizations by repeating the CMIP5/CFMIP-2 AMIP/AMIP + 4K uniform sea surface temperature perturbation experiments with 10 climate models which have had their convective parametrizations turned off. Previous studies have suggested that differences between parametrized convection schemes are a leading source of inter-model spread in cloud feedbacks. We find however that ‘ConvOff’ models with convection switched off have a similar overall range of cloud feedbacks compared with the standard configurations. Furthermore, applying a simple bias correction method to allow for differences in present-day global cloud radiative effects substantially reduces the differences between the cloud feedbacks with and without parametrized convection in the individual models. We conclude that, while parametrized convection influences the strength of the cloud feedbacks substantially in some models, other processes must also contribute substantially to the overall inter-model spread. The positive shortwave cloud feedbacks seen in the models in subtropical regimes associated with shallow clouds are still present in the ConvOff experiments. Inter-model spread in shortwave cloud feedback increases slightly in regimes associated with trade cumulus in the ConvOff experiments but is quite similar in the most stable subtropical regimes associated with stratocumulus clouds. Inter-model spread in longwave cloud feedbacks in strongly precipitating regions of the tropics is substantially reduced in the ConvOff experiments however, indicating a considerable local contribution from differences in the details of convective parametrizations. In both standard and ConvOff experiments, models with less mid-level cloud and less moist static energy near the top of the boundary layer tend to have more positive tropical cloud feedbacks. The role of non-convective processes in contributing to inter-model spread in cloud feedback is discussed.

## 1. Introduction

Equilibrium climate sensitivity (ECS) is a standard measure of the sensitivity of climate models to external forcing, and is defined as the equilibrium change in global mean near-surface temperature following an instantaneous doubling of CO_2_. It remains an important quantity for climate policy, because climate negotiations use the size of the increase in long-term global mean surface temperature as a metric for dangerous anthropogenic interference with the climate system [1]. The Fifth Assessment Report of the Intergovernmental Panel on Climate Change concluded that estimates of the ECS based on observed climate change, climate models and feedback analysis, as well as palaeoclimate evidence indicate a likely range of 1.5–4.5°C [2] and that the dominant source of spread among climate sensitivities from climate models is due to differences in cloud feedbacks, particularly due to low clouds [3].

The Cloud Feedback Model Intercomparison Project (CFMIP) [4] coordinates a number of idealized experiments in CMIP5, which perturb sea surface temperatures and CO_2_ in atmosphere only experiments forced with observed AMIP sea surface temperatures (SSTs) and also in idealized aquaplanet configurations [5]. These experiments include satellite simulators which support quantitative evaluation of clouds using a range of satellite products [6]. Process diagnostics, such as physical temperature and humidity budget tendency terms [7] and high-frequency outputs at selected locations [8], are also included to support the generation and testing of physical hypotheses for cloud feedback mechanisms. CFMIP also coordinates a joint activity on cloud feedbacks with the global atmospheric system study (GASS). The CFMIP–GASS intercomparison of SCM and LES (CGILS) aims to evaluate the performance of global climate model (GCM) physics in single column models (SCMs) using large eddy simulations (LESs) forced consistently in idealized subtropical cloud feedback scenarios associated with well-mixed stratocumulus, stratocumulus over cumulus and shallow cumulus regimes [9,10]. Bretherton [11] provides a review of findings from CGILS and other recent high-resolution cloud feedback studies.

These experiments have led to a number of new studies investigating the physical mechanisms underlying cloud feedbacks, including [7,9,10,12–21]. A number of these studies have implicated parametrized convection (both shallow and deep) as playing a central role in the mechanisms of cloud feedback, although the relative importance of other processes remains unclear. Additionally, a number of studies have employed the so-called ‘emergent constraint’ approach which exploits statistical relationships between observable and predicted quantities across climate models to constrain climate sensitivity [21–25]. These, along with other studies such as [26,27], have tended to find that models with mid-to-high climate sensitivities have more credible simulations of present-day clouds, humidity and convection than those at the lower end of the model range. Some studies have additionally combined the emergent constraint approach with physical arguments [21].

Despite such progress, further work is still required to rigorously test the robustness of the physical mechanisms proposed so far and the constraints that they imply for cloud feedback and climate sensitivity. Given the nature of most model intercomparison projects, multi-model studies can usually only demonstrate that results are consistent with a proposed physical hypothesis. Such hypotheses can be tested more rigorously if we attempt to falsify them using sensitivity experiments. For example, if a particular mechanism is proposed to contribute to positive subtropical feedback, then suppressing the processes involved should weaken that feedback. This process simplification approach has already been applied in some studies with single GCMs [7,14] but has not yet been applied consistently across multiple of climate models; hence, the findings of such studies so far remain highly model specific.

The Selected Process On/Off Klima Intercomparison Experiment (SPOOKIE) is a recent initiative associated with CFMIP which aims to establish the relative contributions of different areas of model physics to inter-model spread in cloud feedback by switching off or simplifying different model schemes or processes in turn. Here, we present results from a pilot study which assesses the impact of convective parametrizations on cloud feedbacks by switching off convective parametrizations in 10 climate models.

Convective parametrizations are generally employed in climate models to represent transports of heat, moisture and momentum associated with convective motions at subgrid scales as well as associated cloud microphysical and precipitation processes [28–30]. Convective parametrizations enable climate models to simulate various properties of atmospheric convection which cannot be accurately represented at the resolved scale, such as allowing moist convection to occur without reaching grid-scale saturation [31]. Previous studies have examined the impact and benefits of parametrized convection in individual models, both by introducing new convective parametrizations [32–35] and by running models with convective parametrizations suppressed [31,36–38]. Additionally, Gettelman *et al*. [39] and Zhao [40] have demonstrated sensitivity of cloud feedbacks to details of convective parametrizations in versions of the NCAR and GFDL models, respectively. These more recent findings (and others discussed below) motivated our choice to focus on the impact of convective parametrizations on cloud feedbacks in this initial pilot study.

Cloud feedbacks could potentially be affected in various different ways by both deep and shallow convective parametrizations. Most obviously, deep convective parametrizations would be expected to influence the formation of cirrus clouds and so could potentially affect cloud feedbacks associated with changes in the properties of high clouds. Although a near cancellation between tropical longwave and shortwave cloud radiative effects (CREs) is observed in regions of deep convective activity where clouds are optically thick [41], such a cancellation is by no means guaranteed in climate models, or in the changing climate. Deep convection schemes could potentially also affect changes in optically thinner cirrus clouds whose impact is mainly in the longwave, by influencing upper tropospheric humidities across the wider tropics. However, as noted above, the dominant source of spread in cloud feedbacks in climate models is due to low clouds. These can potentially be influenced locally by shallow convective parametrizations or by deep convective parametrizations if they trigger in regions where low clouds are prevalent. For example, results from CGILS [10] suggest that the ability of SCMs to correctly diagnose the presence of convection has a substantial impact on low cloud feedback. Zhang *et al*. [10] proposed a mechanism for positive subtropical low cloud feedback in climate models whereby increased entrainment of dry air from the free troposphere into the boundary layer by parametrized convection in the warmer climate reduces low cloud amounts. Alternatively, it is possible that parametrized convection could exert a remote influence on shallow cloud feedbacks in climate models, for example by affecting the temperature and humidity structure of the free troposphere, as suggested by Brient & Bony [14]. Sherwood *et al.* [21] argued that a substantial fraction of the variation in the strength of low level cloud feedback across models is regulated by the strength of ‘lower-tropospheric mixing’ between low- and mid-levels by small-scale parametrized processes such as convection and the resolved large-scale shallow overturning circulation in the present-day climate. These were argued to control the rate at which the boundary layer dries and low cloud reduces as the climate warms. Sherwood *et al.* [21] additionally showed that indirect observable proxies for the lower-tropospheric mixing rate based on the tropical temperature, humidity and vertical velocity in ascending regions were significantly correlated with ECS and cloud feedback, statistically ‘explaining’ just under half of the inter-model variance in the ECS. They also showed evidence of significantly different amounts of low level drying by convective parametrizations between a subset of models in subsiding regions, and suggested that their lower tropospheric mixing mechanism could operate in shallow cloud regions as well as in regions of mean ascent.

Motivated in part by these findings, the pilot SPOOKIE experiments have repeated the CFMIP-2/CMIP5 amip/amip4K experiments with convective parametrizations turned off (convoffamip and convoffamip4K experiments). These experiments are designed to give an indication of the impact of the models’ convective parametrizations on cloud feedbacks, and not of convection in the general sense; convective instability which would be removed by the convective parametrizations in the standard experiments will instead be removed by the models’ turbulent mixing schemes and large-scale dynamics in the ConvOff experiments. If the details of convective parametrizations are indeed responsible for a substantial part of the inter-model spread in cloud feedback, then these experiments might be expected to exhibit a narrower range of cloud feedback. Equally, if parametrized convection is responsible for positive subtropical cloud feedbacks in the GCMs, then the ConvOff experiments would be expected to have neutral or negative cloud feedbacks.

This study is structured as follows. §2 describes the models employed and lists details of the convection schemes and the steps which were taken to switch them off. §3a discusses the impact of switching off convection on the global cloud feedbacks. §3b discusses the impact on cloud feedbacks in various cloud regimes over the low-latitude oceans. In §3c, we discuss the impact on present-day cloud variables and relationships between them and the cloud feedbacks. We discuss the potential role of other processes in contributing to inter-model spread in cloud feedback in §4, and present our overall conclusions in §5.

## 2. Models and experimental design

Our experimental design is based on the CFMIP2/CMIP5 amip and amip4K experiments. The amip experiment forces the atmosphere-only version of the model with observed seasonally and inter-annually varying SSTs and sea ice concentrations, and the amip4K experiment applies a uniform +4 K SST perturbation to the amip experiment [4]. This approach is derived from that of Cess *et al*. [42] which originally diagnosed cloud feedbacks in perpetual July experiments forced with an observed climatology and subject to a uniform +2 K warming. Many of the amip/amip4K experiments used here are pre-existing CMIP5 experiments, but some were run specifically for this intercomparison. These experiments were then repeated with convective parametrizations switched off. Horizontal and vertical resolutions were maintained, but in some cases, other details were changed to maintain the stability of the integrations. A brief description of each model, its convection scheme and the steps taken to switch convection off follows for the various models. Unless stated otherwise below, we use the amip/amip4K experiments from CMIP5. All of the convection off experiments were performed specifically for this study. All experiments were run for 30 years from January 1979 to December 2008, unless stated otherwise below.

CanAM4 [43] has a horizontal resolution of T63 with 35 layers in the vertical. CanAM4 uses a mass flux scheme for deep convection, including aerosol chemistry [44], a prognostic closure based on convectively available potential energy [45] and a parametrization of convective momentum transport [43]. A separate shallow convection scheme is used which is allowed to operate at the same time and location as the deep convection [33]. For the ConvOff experiments, the model was modified, so that it completely bypassed the shallow and deep convection by setting the mass flux to zero.

CESM1-CAM5.1-FV2 [46] was run specifically for this study from UW with a resolution of 1.9° latitude×2.5° longitude and 30 vertical levels. Deep convection within the model is parametrized using a plume ensemble approach with closure based on convective available potential energy as computed for an entraining parcel [44,47]. This parametrization includes momentum transport by convection [48]. The model uses a separate shallow convection parametrization which is formulated with a bulk plume approach and mass-flux closure [49]. The shallow convective parametrization is permitted to operate on all model levels. For the ConvOff experiments, both the deep and shallow convective parametrizations were disabled. Dynamics and physics timesteps for the simulations were shortened from their default values to avoid numerical instabilities.

CNRM-CM5 [50] has a horizontal resolution of 1.4° and 31 levels. The deep convection scheme is described by Bougeault [51] and follows a mass-flux approach. It triggers under conditions related to total (large and subgrid scale) moisture convergence at low levels and vertical conditional instability (CAPE), and the scheme is closed using the Kuo [52] hypothesis. CNRM-CM5 does not have a separate treatment of shallow convection. In the ConvOff experiments, the deep convection scheme was bypassed, and no other changes were required to make the model run.

GFDL-AM2 [53] was run specifically for this study at UNIST with a horizontal resolution of 2.5°×2° and 24 vertical levels. GFDL-AM2 uses the relaxed Arakawa–Schubert convection scheme [54] allowing some modifications documented in [53] (i.e. precipitation efficiency and re-evaporation). There is no special treatment for shallow convection. This was completely switched off in the ConvOff experiments. No other changes were required to make the model run.

GFDL-HIRAM [55] has 50 km horizontal resolution and 30 levels in the vertical, and the amip4K experiment was run specifically for this study. The convection scheme is that of Bretherton *et al*. [56] with additional modifications documented in [55]. It is a mass flux scheme with a single bulk plume which both entrains and detrains. The entrainment/detrainment rate is computed based on buoyancy sorting, which interacts with the environment dynamically and thermodynamically. The amip/amip4K experiments were provided for the 25 year period 1981–2005. All elements of the convection were switched off in the ConvOff experiments, which were provided for the 20 year period 1981–2000.

HadGEM2-A [57] has a horizontal resolution of 1.25° latitude×1.875° longitude and 38 vertical levels. The deep convective parametrization is a mass flux scheme based on Gregory & Rowntree [58] but modified to include a CAPE based closure, convective momentum transport and a simple radiative representation of anvils [59] and more recently an adaptive treatment of detrainment [60]. Shallow convection is treated separately and uses a closure based on [61] with entrainment/detrainment rates as in [62]. Both shallow and deep schemes were switched off in the ConvOff experiments. The timestep was shortened from 30 to 15 min to improve model stability. Additionally, we confirmed that reducing the timestep does not substantially affect the cloud feedbacks in the standard configuration with convection included.

The IPSL-CM5A-LR model has a resolution of 2.5°×1.875° in longitude–latitude, and 39 vertical levels (including eight levels less than 2 km). The physics package of this model version is described in [63,64]. The parametrization of shallow and deep convection is based on Emanuel [65] and modified by Emanuel [66] and Grandpeix *et al*. [67]. This scheme is based on a mass flux representation of adiabatic saturated updraughts and downdraughts, unsaturated downdraughts (driven by re-evaporation of precipitation) and the induced motions of the environmental air. The mixing between cloud and environmental air is based on the ‘episodic mixing and buoyancy sorting’ scheme developed by Emanuel [65]. The simulations with the convection scheme switched off use the same physics, except that the time step (15 min in the original set-up) was reduced by a factor of two (7.5 min) to avoid numerical instabilities. Previous investigations have shown that the model climatology was not significantly dependent on the time step for this range of values. The IPSL-CM5A-LR ConvOff experiments were provided for the 27 year period 1979–2005.

MIROC5 [68] has a horizontal resolution of T85 (1.4°) and 40 levels in the vertical. The convection scheme has a mass flux closure similar to Arakawa–Schubert, but the entrainment rate varies in time and space depending on the temperature and humidity [69]. Shallow convection is not treated separately, but the scheme may represent some shallow cumulus clouds. The entire convection scheme was switched off in the ConvOff experiments. No other modifications were necessary.

MPI-ESM-LR has a horizontal resolution of T63 (which translates to around 200 km grid spacing at the equator) and 47 levels in the vertical. It incorporates modified Tiedtke–Nordeng parametrizations of shallow, deep and mid-level convection [70,71], which are modelled by a unified mass flux formulation with a quasi-equilibrium closure for deep convection, and a moisture closure for shallow convection [72]. All of the above were switched off in the ConvOff experiments, and no other changes were necessary, although the model crashed a few times with high wind speeds. It could in each case however be continued by introducing a small change to the atmospheric state.

MRI-CGCM3 [73] has a resolution of (T159, L48). The Yoshimura cumulus scheme [74] is a mass flux spectral cumulus parametrization scheme that explicitly considers an ensemble of multiple convective updrafts. This cumulus scheme has the advantages that the variables in entraining and detraining convective updrafts are calculated in detail layer-by-layer as in the Tiedtke scheme, and that a spectrum of convective updrafts with different heights owing to difference in entrainment rates is explicitly represented, as in the Arakawa–Schubert scheme. No shallow convection scheme is used, and the Yoshimura cumulus scheme is designed to reproduce all depths of convection. For the ConvOff experiments, all elements related to convection scheme were switched off. No additional changes were required to make the model run stably without convection.

## 3. Results

### (a) Global mean cloud feedbacks

**Figure 1.**
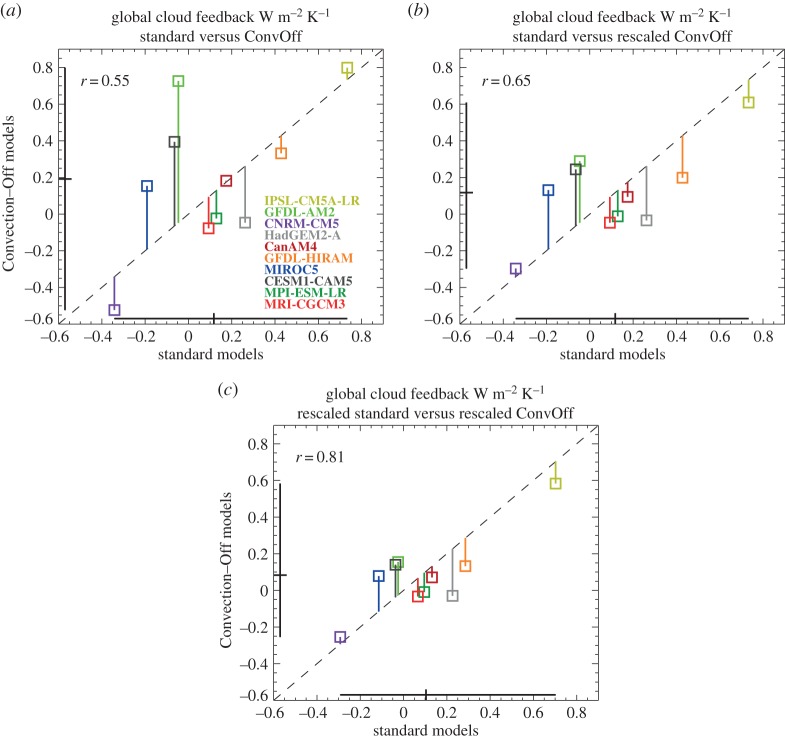
(*a*) Global mean net cloud feedbacks in CFMIP amip/amip4K experiments and convoffamip/convoffamip4K experiments without parametrized convection. This is diagnosed as the change in the global mean net cloud radiative effect (CRE) between the amip and amip4K experiments, normalized by the global mean near-surface temperature response and includes the effects of climatological cloud masking on the non-cloud feedbacks. Black lines denote the ranges in the values and the diagonal line indicates the one-to-one line. The lengths of the vertical coloured lines indicate the differences between standard and ConvOff values for the individual models. The linear correlation coefficient r is also shown. Panel (*b*) shows the same but with the ConvOff feedbacks rescaled by the factor required to bring the global mean net CRE in the convoffamip experiment into agreement with the standard amip experiment. Panel (*c*) shows the result of scaling all feedbacks by the factors required to bring their control experiments into agreement with an observed value of the net CRE (−17.1 W m^−2^).

Figure 1*a* shows a scatterplot of the global mean cloud feedbacks in the 10 models examined with and without convective parametrization. These cloud feedbacks are diagnosed using the commonly employed method of taking the change in the long-term annually averaged global mean net CRE between the amip and amip4K experiments for all available years (as documented in §2) and dividing by the corresponding change in the long-term annually averaged global mean near-surface temperature [75]. This method tends to yield less positive/more negative values of cloud feedback than alternative approaches based on the alternative ‘partial radiative perturbation’ method because it includes the masking effect of climatological cloudiness on non-cloud feedbacks [76]. It is however a good predictor of inter-model spread in cloud feedback [77].

By comparing the models’ global cloud feedbacks with and without convective parametrization, we can directly test the hypothesis that a substantial fraction of the inter-model spread is due to differences in the details of the convective parametrizations. If this were the case, then we would expect to see a considerable reduction in the inter-model spread in the ConvOff experiments. The standard models have a range of 1.07 (−0.34 to 0.73) W m^−2^ K^−1^. This range is not reduced however in the ConvOff experiments; in fact it increases by 23% to 1.32 (−0.52 to 0.80) Wm^−2^ K^−1^. Similarly, the standard deviation increases by 25%. At face value, this would seem to indicate that differences in the details of convective parametrizations are not the dominant cause of inter-model spread in global mean cloud feedbacks in this particular selection of climate models.

Before drawing firm conclusions on this point however, we consider an alternative potential explanation for this result. Previous studies which have examined the impact of shallow cumulus parametrizations in models have shown that the introduction of shallow convection schemes tends to reduce cloud in the boundary layer [32–35]. It is, in principle, possible that enhanced cloudiness in the ConvOff experiments could in itself have an impact on the cloud feedbacks which would not be present if all of the models were retuned to have similar amounts of cloud as the standard configurations. If this effect was to vary between the models, it could, in principle, inflate the inter-model spread in the feedbacks and offset a reduction in spread associated with the removal of differences between the convection schemes. For example, if switching off convection in a model were to say double the amount of cloud in the amip and the amip4K experiment, then that would imply a doubling of the difference between them—i.e. a doubling of the cloud response. Such a scenario would imply a relationship between the amount of cloud in the control simulation and the strength of the cloud response to climate change, as has been proposed by Brient & Bony [13]. They found a relationship between the amount of subtropical low level cloud and the magnitude of its change in the warmer climate across different versions if the IPSL GCM. This behaviour was interpreted in terms of a ‘beta feedback’ between low level cloud fraction, the low clouds’ longwave radiative cooling and the relative humidity of the boundary layer. Stronger coupling between these quantities was argued to result in a larger low level cloud fraction and an amplification of any change in low cloud fraction in the warmer climate. If such relationships are present in the models more generally, then it might be possible to estimate what the low level cloud response would be in the ConvOff models if they were tuned to agree with the standard versions. This could for example be achieved by scaling the convoffamip and convoffamip4K low level cloud fractions by the factor required to bring the convoffamip low cloud fraction into agreement with the amip value—i.e. dividing them both by the low cloud fraction from convoffamip and multiplying by that from the standard amip experiment. This would effectively scale the ConvOff cloud response by the same factor. Equivalently, we can estimate approximately what the global mean cloud feedback would be following a retuning by taking the global mean net cloud feedback in the ConvOff experiments and scaling that by the ratio of the global mean net CRE from the amip experiment to that from convoffamip. Figure 1*b* shows the relationship between the cloud feedbacks in the standard experiments as in figure 1*a* versus rescaled ConvOff feedbacks calculated in the manner described above, to give an estimate of the spread in the ConvOff feedback making an allowance for the effect of changes in the present-day net CRE. This results in a range of 0.91 (−0.30 to 0.61) W m^−2^ K^−1^ for the scaled ConvOff feedbacks, a reduction of 31% compared with their original range of 1.32 (−0.52 to 0.80) W m^−2^ K^−1^. Similarly, the standard deviation is reduced by 38%. This constitutes a modest reduction of 15% compared with the range of the standard models (1.07), but not a substantial one. Similarly, the standard deviation reduces by 22%. This suggests that even if the ConvOff experiments were re-tuned to bring their control simulations into closer agreement with the standard model versions, the overall range in their cloud feedbacks would not be greatly reduced, supporting our initial conclusions above.

We should of course bear in mind the fact that this estimate of the impact of retuning is very simplistic and could be inaccurate. However, there are reasons to be optimistic. First, most of the ConvOff feedback estimates are closer to the standard ones after they are rescaled. The points in figure 1*b* are mostly closer to the diagonal line than those in figure 1*a*, and the correlation coefficient between standard and ConvOff cloud feedbacks increases from 0.55 to 0.65 with the rescaling, becoming significantly different from zero at the 5% confidence level. This is what we would expect to see if (i) the scaling method was correctly adjusting for the effects of increased low level cloudiness on the cloud feedbacks in the ConvOff experiments and (ii) such impacts were contributing substantially to the differences between the cloud feedbacks in the standard and ConvOff experiments. Additionally, we find that if all of the feedbacks (standard and ConvOff) are rescaled to values consistent with the observed net CRE value of −17.1 W m^−2^ from the CERES EBAF (Clouds and Earth’s Radiant Energy Systems Energy Balanced and Filled) dataset [78], then the correlation increases even further to 0.81 (figure 1*c*). This suggests that the rescaling is generally bringing the standard and ConvOff feedbacks into closer agreement, which would only be expected if the rescaling approach was working effectively. Here again the rescaled ConvOff experiments have only a slightly smaller spread than the standard experiments (a reduction again of 15% in the range and 22% in the standard deviation). It is also interesting to note that rescaling the standard experiments to have the same global mean net CRE reduces the range in their global cloud feedbacks slightly by 8% and the standard deviation by 13%, which suggests that a small part of the spread in the standard experiments might be attributable to differences in present-day cloud biases.

Although the impact of parametrized convection on the overall range is relatively small (a reduction of 15% allowing for changes in present-day CRE), we note that larger impacts are present in some models which do not affect the overall range in this particular ensemble. The largest impact of turning off convection is seen in GFDL AM2, in which the net cloud feedback increases from −0.05 to 0.75 W m^−2^ K^−1^, an increase of 0.8 W m^−2^ K^−1^. This is substantial compared with the overall range in the standard experiments of 1.07 W m^−2^ K^−1^. However, this model has the largest increase in global mean net CRE in the control (−31.9 in amip compared with −80.1 W m^−2^ in convoffamip). Once the GFDL AM2 ConvOff feedback is rescaled by the factor 31.9/80.1, it becomes 0.3 W m^−2^ K^−1^, just 0.35 W m^−2^ K^−1^ larger than the standard GFDL AM2 feedback. This change is now considerably smaller than the overall cloud feedback range of 1.07 W m^−2^ K^−1^. Turning off parametrized convection can have substantial impacts on the global cloud feedback if the net CRE in the control simulation is allowed to change substantially, but in the models examined here, this effect is considerably smaller in models where the net CRE in the control does not change substantially, or where the effects of changing the present-day CRE are taken into account. It is also, in principle, possible that making different changes to the details of convective parametrizations which are not included in our current ensemble could have larger impacts on global cloud feedbacks than those seen here. Suppressing the convection schemes in a particular set of models tells us about the impact of the structure and parameter settings of the convection schemes in those models, and not the impact of all possible convection schemes or parameter settings, which might have more extreme impacts. Previous studies with individual models have in some cases indicated that changing parameter values in convection schemes can have a substantial impact on ECS. For example, Rougier *et al*. [79] show that weakening lateral entrainment in the convection scheme in HadSM3 increases the climate sensitivity substantially. However, Joshi *et al*. [80] found that that the high sensitivity in HadSM3 on reducing entrainment is attributable to a strong stratospheric water vapour feedback, and that the impact on the cloud feedback is small. It is also important to note that our current standard and ConvOff ensembles already span the range in cloud feedbacks typically seen in climate models [75]. Hence, adding new models or different convection schemes to our current ensembles would not affect our finding that the models can explore the full range of contemporary cloud feedbacks without parametrized convection.

In summary, our conclusion is that while parametrized convection influences the strength of the cloud feedbacks substantially in some models, differences in convection schemes between the models do not have a substantial impact on the overall range in global cloud feedbacks in the models examined here. The models are capable of exploring much of the overall range in feedbacks without convective parametrizations active, indicating that other aspects of model formulation are equally important in determining the overall range of cloud feedback.

### (b) Cloud feedbacks over the low-latitude oceans

Many studies have highlighted the dominant role of the low-latitude oceans in contributing to inter-model spread in cloud feedback [77,81,82]. Such studies have also identified a dominant role for shallow cloud feedbacks over the tropical oceans by sorting the model responses into shallow versus deep cloud regimes using quantities such as 500 hPa vertical velocity or lower-tropospheric stability (LTS), the latter quantity being defined as the difference in potential temperature of the air at 700 hPa and at the surface [83]. Medeiros & Stevens [84] classified tropical clouds using joint distributions of these two variables, finding that vertical velocity separates regimes dominated by boundary layer clouds from those associated with higher and/or deeper clouds, whereas LTS is more effective at separating shallow cumulus and stratocumulus within shallow cloud regimes. They also noted that precipitation and 500 hPa vertical velocity are similarly effective in identifying regions of tropical convection. We have experimented with various compositing approaches for this study, and have developed a single hybrid index based on precipitation and LTS which aims to combine the benefits of these two indices. We chose precipitation, because we consider this to be a more robust indicator of the strength of tropical moist convection than, say, the vertical velocity at 500 hPa, which will be more sensitive to the profile of the resolved vertical motion.

**Figure 2.**
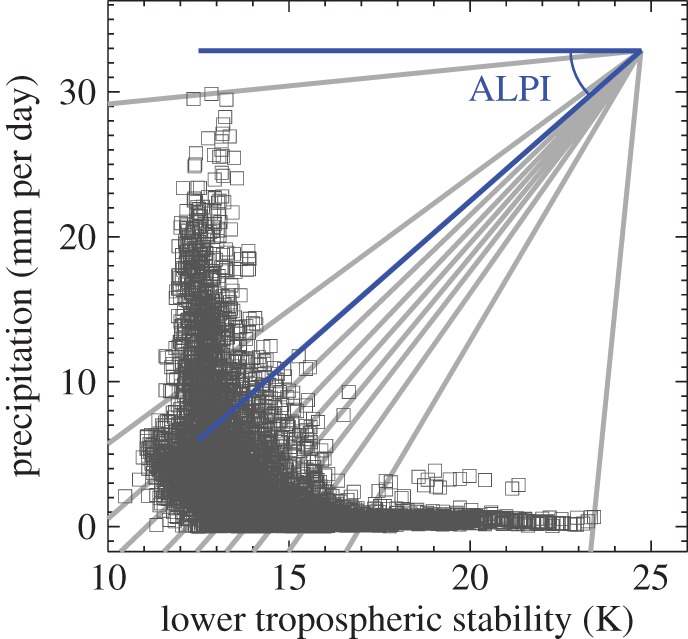
Scatterplot of LTS and precipitation from the HadGEM2-A amip experiment for February 1979 over the low-latitude oceans (30°N/S). The angular LTS/precipitation index (ALPI) is diagnosed as the angle of declination of a line connecting each point in LTS/precipitation space with an ‘anchor point’ on the top right. Locations with the strongest precipitation rates give values of ALPI of around 5°, whereas locations with the largest values of LTS result in an ALPI value of around 85°. Grey lines indicate the boundaries of ALPI percentile bins each covering 10% of the low-latitude ocean area.

Figure 2 shows a scatterplot of precipitation against LTS for a single monthly mean which serves to illustrate the difficulties of using either variable alone to characterize the joint distribution. Much of the variation in LTS occurs in a narrow range of weakly precipitating regimes and so cannot be captured by a precipitation based index, whereas much of the variation in precipitation occurs in a narrow range of weak LTS values. The angular LTS/precipitation index (ALPI) is designed to sample the joint distribution of both variables and is calculated using the following procedure. An ‘anchor point’ is formed near the location [LTS_max_, *P*_max_] which appears on the top right of the scatterplot in figure 2. The normalized distances of each LTS and precipitation value from this anchor point are then calculated thus:
3.1
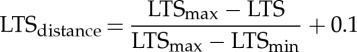
and
3.2
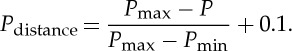
ALPI is then diagnosed as the angle of declination in degrees of the line taken between the data point and the anchor point in normalized LTS/precipitation space:
3.3
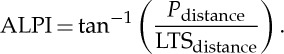
The values of 0.1 are added to move the anchor point slightly, thus avoiding division by zero for the largest values of LTS while treating LTS and precipitation symmetrically. This reduces the range of ALPI values taken from 0–90° to 5–85°.

**Figure 3.**
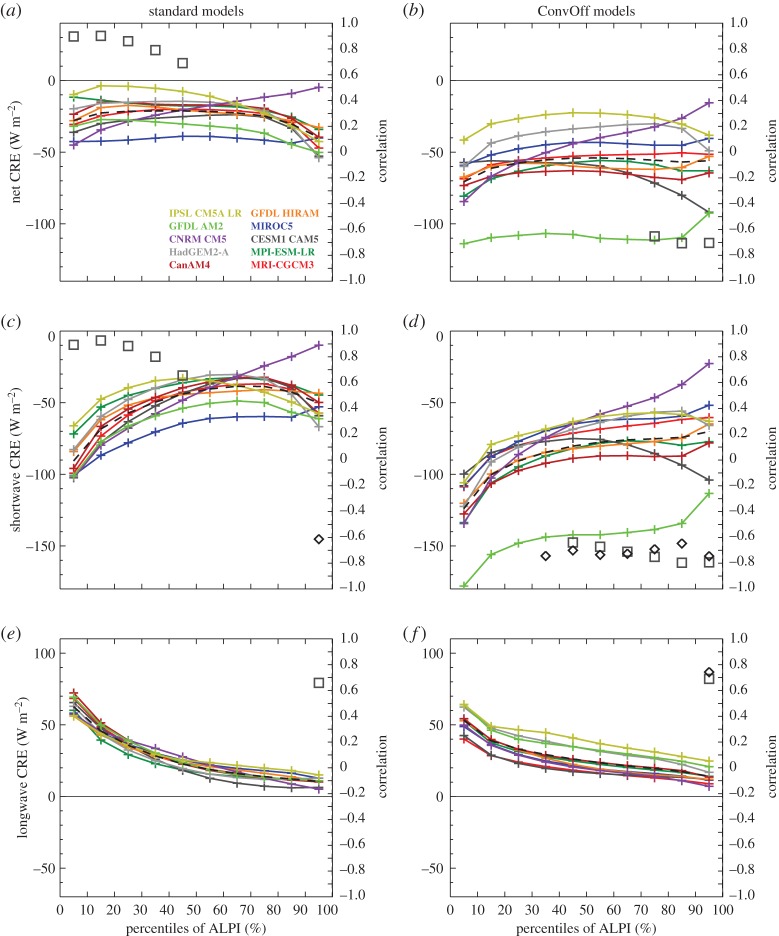
Composites of net, shortwave and longwave cloud radiative effect (CRE) over low-latitude oceans (30°N/S) in the amip control experiments (*a*,*c*,*e*) and convoffamip (*b*,*d*,*f*), sorted by percentiles of the angular LTS/precipitation index (ALPI). Black diamonds denote correlations with the net cloud feedback in the same ALPI bin which are significant at the 95% level. Squares indicate a significant correlation with the values in the bin and the average of the net cloud feedback over the entire low-latitude ocean domain. Ensemble mean values are shown with a black dashed line.

**Figure 4.**
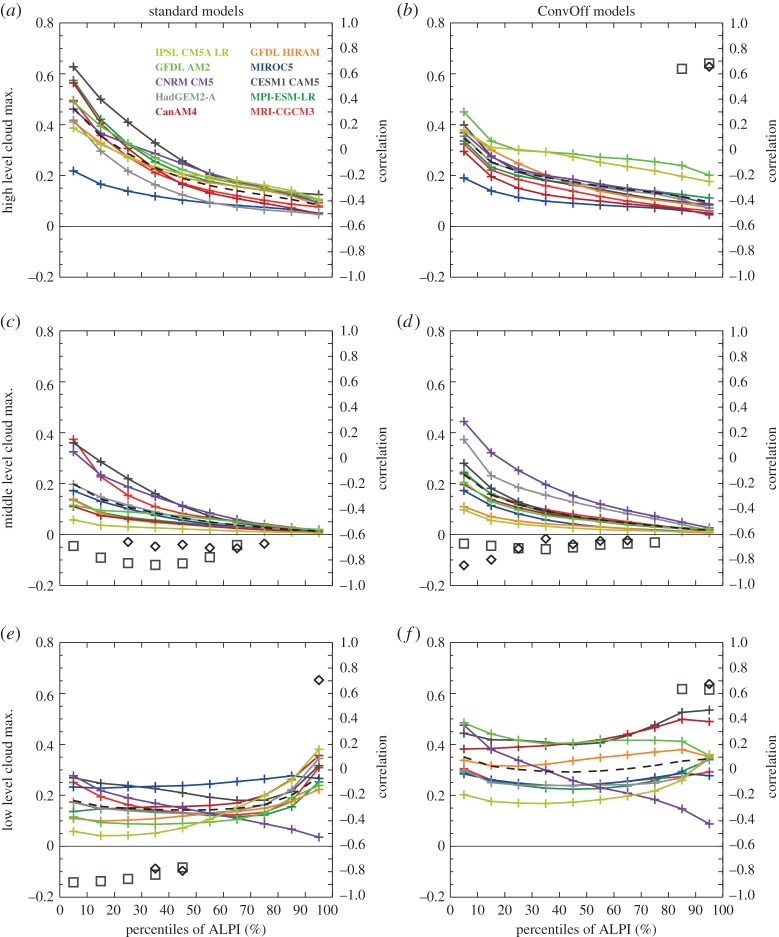
As figure 3 but for maximum low, mid and high cloud fractions. These are dimensionless, taking values between 0 and 1, and are diagnosed from profiles of monthly mean cloud fraction on model levels by taking the maximum values in the pressure ranges 0–440, 440–680 and 680 hPa–surface.

**Figure 5.**
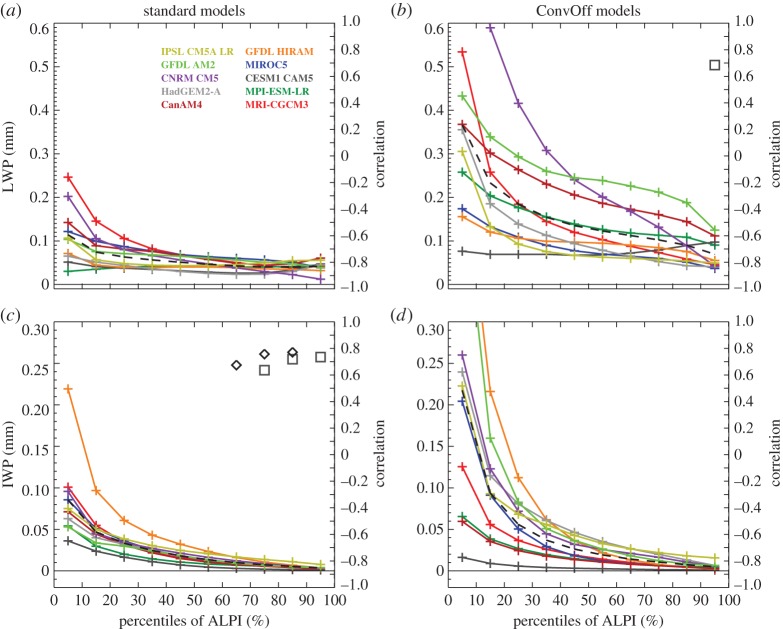
As figure 3 but for liquid water path (LWP) and ice water path (IWP). The compressed ranges of the LWP and IWP scales are chosen to support comparison of the smaller values, but by necessity exclude the 0–10th percentile values of LWP for the CNRM-CM5 convoffamip experiment (1.0 mm) and the 0–10th percentile values of IWP for GFDL AM2 and HIRAM convoffamip experiments (0.47 and 0.51 mm, respectively).

Figure 3 shows composites of present-day CRE over the low-latitude oceans in the standard and ConvOff experiments, sorted into area-weighted percentiles of ALPI. These are calculated using monthly means for all years available in each experiment (see §2), by sorting each month by ALPI percentiles and averaging the results in each bin in time. Percentiles are used to ensure that the individual bins each cover one tenth of the total area, following the approach of Wyant *et al*. [85]. The areas contributing to each of the bins will thus be the same in the present and future climate, removing any need to take account of changing bin populations as necessary when using fixed intervals. The 0–10% percentile range of ALPI includes the tenth of the tropical ocean area with the strongest precipitation, and captures the strongest values of the longwave CRE in each of the models (figure 3*e*,*f*) as well as the largest upper-level cloud fractions (figure 4*a*,*b*) and ice water paths (figure 5*c*,*d*). Meanwhile, the 80–100% ALPI range covering the strongest regimes of LTS includes the local maxima in low-level cloud fractions (figure 4*e*,*f*) and minima in the net CRE (figure 3*a*,*b*) present in many of the models and in the ensemble mean.

**Figure 6.**
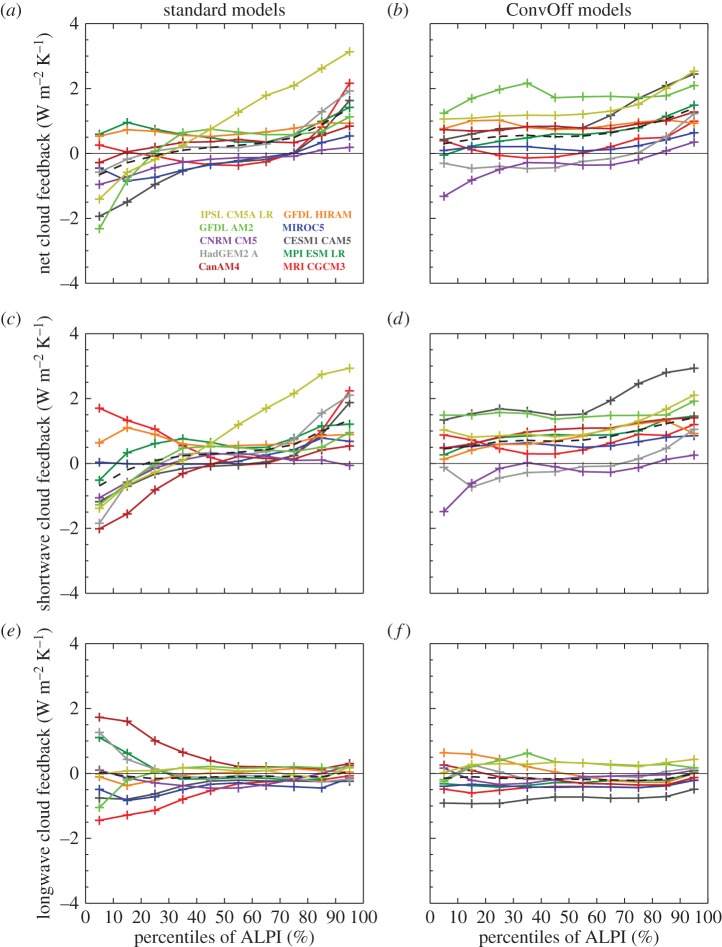
Composites of net, shortwave and longwave cloud feedback over low-latitude oceans (30°N/S) in the amip/amip4K experiments (*a*,*c*,*e*) and convoffamip/convoffamip4K experiments (*b*,*d*,*f*), sorted by percentiles of the angular LTS/precipitation index (ALPI). Regions of strongest precipitation associated with deep convection fall in the lower percentiles while regions of strong static stability where shallow clouds predominate fall into the higher percentiles. The black dashed line shows the ensemble mean values in each bin.

Figure 6 shows equivalent composites of the cloud feedbacks. These are diagnosed by sorting the net CRE in the amip and amip4K experiments into percentiles of ALPI, taking the difference in each bin and then dividing by the global mean change in near-surface temperature. This diagnosis of the cloud feedback will include the effects of cloud masking as discussed above. Shortwave cloud masking is negligible over the tropical oceans, but the longwave component is expected to contribute up to −1 W m^−2^ K^−1^ to the longwave and net CRE responses in the subsiding regions of the tropics and up to −2 W m^−2^ K^−1^ in deep convective regions (see [86] and its fig. 10). In the warmer climate, both LTS and precipitation increase on average across the tropics; the position of the anchor point is tied to the maximum LTS and precipitation values, and the equally sized ALPI bins continue to sample comparable sections of the tropical cloud regime distribution; for example, the 0–10% percentile bin continues to include the 10% of the tropical ocean area with the strongest precipitation.

Figure 6*c* shows that the standard models have largely positive shortwave cloud feedbacks in the 40–100th percentile range of ALPI, where shallow clouds are expected to dominate the cloud feedbacks. Based on analysis of cloud feedbacks in single column versions of several GCMs, Zhang *et al*. [10] proposed a mechanism for positive subtropical feedback in climate models whereby increased entrainment of dry air from the free troposphere into the boundary layer by parametrized convection in the warmer climate reduces cloud. Additionally, Sherwood *et al*. [21] argued that enhanced small-scale lower-tropospheric mixing of moisture by parametrized processes such as convection in the warmer climate contributes (along with other factors) to positive low cloud feedbacks in models. The ConvOff experiments should provide an indication of the relative importance of these processes in the full models; if, for example, the dominant cause of the positive cloud feedback in the models was due to the action of the parametrized convection schemes, then we would expect to see substantial reductions in this feedback’s magnitude in the ConvOff experiments. Comparison of the standard and ConvOff feedbacks in figure 6 indicates that this is not generally the case. The ensemble mean net and shortwave cloud feedbacks are if anything slightly more positive in the 40–100th percentile ranges. The magnitude of the positive subtropical cloud feedback is reduced slightly in some cases (e.g. in IPSL-CM5A-LR, HadGEM2-A and CNRM-CM5) but still remains positive in the 80–100th percentile range where stratocumulus clouds are expected to dominate the cloud feedback. These results indicate that processes other than parametrized convection are largely responsible for positive subtropical cloud feedback in the climate models examined here. For example, Zhang *et al*. [10] also suggest that enhanced cloud top entrainment by the models’ boundary layer schemes might contribute to positive cloud feedback, whereas Sherwood *et al*. [21] argue that enhanced lower-tropospheric mixing by resolved shallow circulations will also contribute.

Sherwood *et al*. [21] also argue that inter-model differences in the strength of small-scale lower-tropospheric mixing by parametrized convection contribute to the spread in the low-level cloud feedback in models. If this was a substantial effect in our ensemble, then we might expect to see a reduction in the range in net and shortwave cloud feedback in the mid–upper ALPI range. However, our results show that the inter-model range in net and shortwave cloud feedback is not greatly changed in the upper ALPI range (where stratocumulus clouds are expected to dominate the feedbacks), whereas it actually increases slightly in the mid ALPI range where we expect shallow cumulus clouds to dominate.

Figure 6*e*,*f* does however show evidence for convective parametrizations making a substantial contribution to inter-model spread in one aspect of cloud feedback; namely that of the longwave cloud feedback in strongly precipitating regions of the tropics in the 0–30th percentile range. The range of this feedback is substantially reduced in the ConvOff experiments, indicating a considerable local contribution from inter-model differences in the details of convective parametrizations. We also considered the possibility that the reduction in spread was caused by changes in water vapour and/or lapse rate feedbacks via their cloud masking contributions to the change in longwave CRE; we ruled this out, however, as the reduction in spread is also clearly seen in the outgoing longwave radiation response but not the clear-sky equivalents (not shown). In contrast to the strongly precipitating regimes, the inter-model range in longwave cloud feedback increases slightly in the 50–100th percentile ALPI range in the ConvOff experiments, which could be a consequence of increased diversity in properties of the models’ cirrus clouds in the absence of retuning.

### (c) Impact of convective parametrization on present-day cloud variables and relationships with cloud feedbacks

Here, we discuss the impact of convective parametrizations on various cloud variables in the present-day simulations, and their relevance to the cloud feedbacks. Many studies have identified statistically significant relationships between climate model predictions of climate sensitivity or cloud feedback and aspects of their present-day simulations which are, in principle, observable [21–25]. The use of such relationships to place observational constraints on climate predictions from models has recently come to be known as the ‘emergent constraint’ approach [87]. Caldwell *et al*. [88] identify a number of potential pitfalls with this approach, and argue that such ‘data mining’ approaches are best used to identify potential relationships which are then validated or discarded using physically based hypothesis testing. Sherwood *et al*. [21] is one of the relatively few studies of this type which develops and interprets such constraints in conjunction with testable physical arguments. In this section, we identify a number of statistically significant relationships between present-day cloud properties within ALPI regimes and the net cloud feedbacks within those regimes and also averaged over the entire low-latitude ocean area. Our results are used to motivate the following discussion of potential physical processes or mechanisms other than those associated with convective parametrization which might be contributing to inter-model spread in cloud feedback, and how such ideas could be tested via further sensitivity experiments in the future. Note that in this study we mainly focus on relationships between cloud properties and cloud feedbacks within regimes, and do not attempt to find relationships with overall climate sensitivity, the spread of which depends on other factors as well as cloud feedback [77,82]. The discussion below focuses on relationships which appear in both ensembles; we consider correlations which are present in one ensemble or the other but not both unlikely to be robust or relevant to the processes explaining the overall spread in cloud feedback.

We return to figure 3 which shows composites of the present-day CRE from the models. The near cancellation between tropical longwave and shortwave CRE observed in regions of deep convective activity where clouds are optically thick [41] is not reproduced by a number of the standard models (for example in the 0–30th percentile ALPI range), and this is exacerbated in the ConvOff experiments. These generally have more negative shortwave and net CRE values across the tropics, in line with our expectation of increased low-level cloudiness. Figure 4 confirms that low-level cloud fraction is larger as expected, although figure 5 additionally indicates considerably larger grid-box mean liquid water paths (LWPs) in the ConvOff models compared with the standard configurations, which may additionally contribute to the larger magnitude of the net and shortwave CRE. This is particularly notable in GFDL AM2, which has relatively large values of both low cloud fraction and LWP in its ConvOff experiment.

The ConvOff models also have a tendency for increased values of longwave CRE in the 40–100th percentile range (figure 3*e*,*f*) which we attribute to a combination of increased high-level cloud fraction (figure 4*a*,*b*) and ice water path (IWP; figure 5*c*,*d*). One possible explanation for this might be that, in the absence of convective parametrization, cloud condensate is rained out less efficiently in deep convective regions, and this increases cirrus outflow into the surrounding regions in the ConvOff experiments. The ConvOff experiments show larger cloud liquid and ice water paths in strongly precipitating regions, consistent with this idea (figure 5).

The ConvOff models also show a larger spread compared with the standard models in longwave CRE (figure 3*e*,*f*), high-level cloud fraction (figure 4*a*,*b*) and IWP (figure 5*c*,*d*), which may contribute to the slightly larger spread in longwave cloud feedback in the 40–100th percentile range discussed in the previous section.

Figure 3 also shows correlations between the CRE values for the models in each ALPI bin and the net cloud feedback in that bin (diamonds) and also the net cloud feedback averaged over the entire low-latitude ocean area covered by the 10 ALPI bins (squares). These are plotted only if they are statistically significant at the 95% level, as determined by the resampling bootstrap method [89], sampling with replacement 10 000 times. In the ConvOff experiments, the shortwave CRE in the 40–100th percentile range is significantly anti-correlated with the net cloud feedback, both within the equivalent ALPI bins and across the low-latitude oceans. Because the GFDL AM2 ConvOff experiment is an outlier, we checked to see whether these correlations were mainly reflecting the unusual behaviour of this one model by repeating the calculation without it. With GFDL AM2 removed, the ConvOff experiments show similar correlations, but confined to the 80–100th percentile range only for the shortwave CRE only (not shown), much like that seen in the standard experiments in figure 3*c*. Hence, both ensembles hint at a tendency for the models with the largest magnitudes of the shortwave CRE in the most stable cloud regimes to have more positive cloud feedbacks in those regimes. This provides support for the argument of Brient & Bony [13] discussed above, in which models with more low level cloud tend to have more positive feedbacks. Additional support for this argument is provided by the fact that the low cloud fractions in the 90–100th percentile range are positively correlated with the net cloud feedbacks in the same range (figure 4*e*,*f*).

Figure 3*e*,*f* also shows that both the standard and ConvOff ensembles have positive correlations between the values of the longwave CRE in the 90–100th percentile range and the cloud feedback averaged over the low-latitude oceans, indicating that models with stronger longwave CRE tend to have more positive cloud feedbacks. Figure 4*b* and 5*c* show similar correlations between the low-latitude ocean cloud feedback and high cloud fraction in the ConvOff ensemble and IWP in the standard models, respectively.

Additionally, both ensembles have mid-level cloud fractions which are anti-correlated with the low-latitude ocean cloud feedback and also with the local net cloud feedback over much of the 0–70th percentile range (figure 4*c*,*d*). The robustness of the anti-correlation between mid-level cloud and cloud feedback across the two ensembles, combined with the large area over which such correlations are present, suggests that the processes controlling mid-level cloudiness in the tropics should be considered in any arguments put forward to explain the mechanisms of inter-model spread in cloud feedbacks over the tropical oceans.

**Figure 7.**
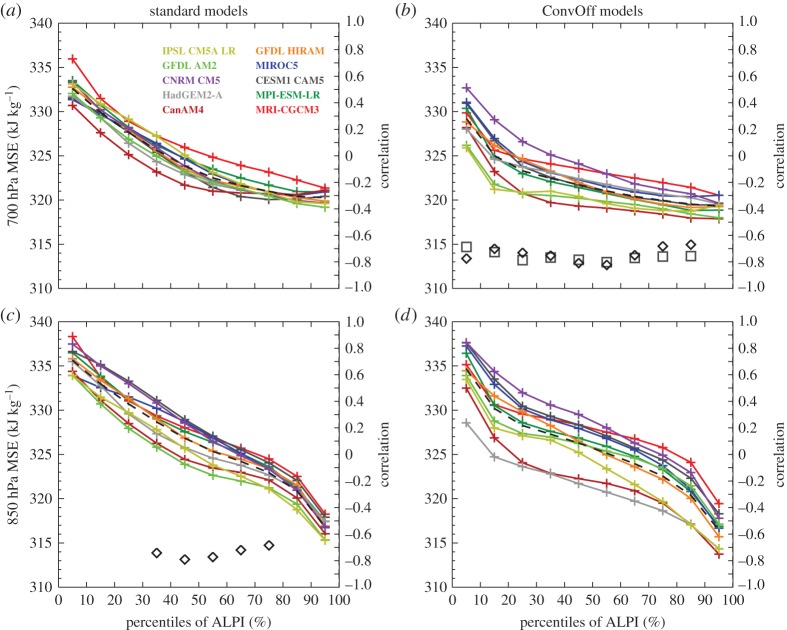
As figure 3 but for moist static energy (MSE) at 700 and 850 hPa.

We also examined ALPI composites of a number of other quantities including measures of vertical gradients in temperature, humidity and subsidence rate as in Sherwood *et al*. [21] (not shown). These indices did not show robust correlations with feedbacks across both of our ensembles and so we do not discuss them further here. We also examined the moist static energy (MSE), a thermodynamic quantity which measures the total energy in a parcel of air, including sensible heat owing to temperature, latent heat owing to water vapour and potential energy owing to height, which is defined as

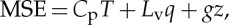
where *C*_p_ is the specific heat of air at constant pressure, *T* is temperature, *L*_v_ is the latent heat of vaporization, *q* is the specific humidity, *g* is the acceleration owing to gravity and *z* is the height above the surface. Source terms for MSE in the atmosphere include surface sensible and latent heat fluxes and absorption of solar radiation, whereas longwave radiative cooling is the major sink term. MSE is redistributed within the atmosphere in the vertical by convective and turbulent mixing processes, and both vertically and horizontally by the large-scale atmospheric circulation. Because MSE is conserved during phase changes between water vapour and liquid water associated with cloud condensation and evaporation of clouds and precipitation it is a convenient indicator of heat transport within the atmosphere in the presence of clouds and precipitating moist convection [31,90]. MSE budgets have more recently been used in studies examining the influence of changes in large-scale advection, convective and turbulent mixing on cloud feedbacks [7,14]. Figure 7 shows that significant anti-correlations are present between the models’ cloud feedbacks and their present-day values of the MSE in the lower troposphere, which is consistent with the argument that the overall spread in cloud feedback is regulated by processes associated with lower-tropospheric mixing, as proposed by Sherwood *et al*. [21]. In the standard models, the correlations are seen with the MSE at 850 hPa, a level chosen by Sherwood *et al*. [21] to represent thermodynamic properties near the top of the boundary layer. Similar correlations are present in the ConvOff models at the slightly higher level of 700 hPa, usually considered to be more representative of the lower free troposphere. It is however possible that in the absence of parametrized convection, turbulent mixing plays more of a role in transporting water vapour into the lower free troposphere and in doing so deepens the boundary layer to have a top closer to 700 hPa. The possibility of deeper boundary layers in the ConvOff experiments is supported by a tendency for slightly larger mid-level (440–680 hPa) cloud fractions in the ConvOff experiments (figure 4). The presence of relationships between low level MSE and cloud feedback in both standard and ConvOff models again suggests that inter-model differences in parametrized convection are not the dominant cause of overall spread in the cloud feedbacks in these models, and that other processes are largely responsible. We discuss such possibilities further below.

## 4. Discussion

Our results indicate that inter-model differences in the details of convective parametrizations cannot explain the overall range in cloud feedbacks in the models examined here; the models exhibit comparable spread in cloud feedback when their convective parametrizations are switched off. Here, we discuss other processes which could be contributing to inter-model spread in cloud feedback, and suggest some ways in which such ideas could be tested using further process simplification experiments in future work.

Sherwood *et al.* [21] and Zhao [40] both argue that precipitation efficiency plays an important role in cloud feedback. Sherwood *et al*. [21] define precipitation efficiency in terms of the amount of precipitation for a given vertical transport of water vapour from the boundary layer to the free troposphere. These transports are associated with ‘lower-tropospheric mixing’ by small-scale processes such as convection or turbulence which are parametrized in GCMs, and by large-scale mixing associated with resolved motions. Sherwood *et al*. [21] argue that models with stronger lower-tropospheric mixing will have a stronger drying of the boundary layer, and that this effect will strengthen in the warming climate and will reduce low-level cloud, resulting in a positive low cloud feedback. Our finding that models with more positive cloud feedbacks tend to have less MSE near the top of the boundary layer in the present climate is consistent with this argument; stronger lower-tropospheric mixing in the present climate in higher sensitivity models could deplete boundary layer MSE more by transporting it from the boundary layer to the free troposphere at a faster rate. Our findings suggest however that processes other than parametrized convection are required to explain the overall range of cloud feedbacks in the models examined here.

Zhao [40] makes a distinction between convective precipitation efficiency associated with parametrized convection and a large-scale precipitation efficiency associated with the stratiform cloud and precipitation schemes in the models. In the absence of convective parametrization, lower-tropospheric mixing must be achieved by resolved motions or by small-scale mixing by the models’ parametrized turbulence schemes. If the strength of such mixing is not regulated by inter-model differences in precipitation efficiency arising from the differences in the models’ convection schemes, it might instead be regulated in a similar way, but by inter-model differences in the precipitation efficiency associated with other parts of the model formulation. The precipitation efficiency, defined as the amount of surface precipitation for a given vertical transport of water vapour from the boundary layer to the free troposphere, could depend on various aspects of model formulation, including cloud parametrizations, cloud precipitation microphysics and their interactions with turbulent mixing and entrainment. Models which form precipitating clouds easily at mid-levels in the tropics will rain out to the surface efficiently for a given upward transport of water vapour. Conversely, models that form clouds and condensate less easily at mid-levels in the tropics might instead need to produce condensation and latent heat release at higher levels in order to balance atmospheric radiative cooling in the tropical free troposphere. Precipitation falling from clouds which are higher in the atmosphere will be more likely to evaporate before reaching the surface, producing more evaporative cooling to offset the latent heat release provided by cloud condensation. Such models might thus require a larger upward transport of water vapour by lower-tropospheric mixing to maintain a given net latent heat release and surface precipitation rate, compared with models which are able to condense more easily at mid-levels and so rain out more efficiently to the surface. Hence, models with less mid-level cloud might have weaker precipitation efficiencies and need to transport more water vapour vertically by lower-tropospheric mixing, drying the boundary layer more than models with more mid-level cloud. Such an effect would be expected to strengthen proportionally with the hydrological cycle as the climate warms and the total amount of atmospheric radiative cooling, net latent heat release and surface precipitation increase, resulting in models with less mid-level cloud having stronger positive low-level cloud feedbacks. Such arguments could potentially explain the anti-correlation between mid-level cloud fractions and cloud feedbacks seen in both ensembles examined here.

We note that these arguments rely on cloud fraction at a given level being a useful proxy for condensation rate and precipitation efficiency; although these quantities are not necessarily exactly equivalent, a relationship between them is clearly plausible in that a model with a larger cloud fraction for a given in-cloud condensation rate will have a larger grid-box mean condensation rate, and hence a stronger condensate source term to support precipitation. We also note that inter-model differences in mid-level cloud fraction and precipitation efficiency may ultimately be due to a range of model formulation differences, including model representations of turbulent mixing and entrainment, which can have effects in the free troposphere as well as the boundary layer, as demonstrated by Tsushima *et al*. [91]. The depth of the shallow circulation discussed by Sherwood *et al*. [21] could also influence the amount of mid-level cloud and the bulk precipitation efficiency; for example, a model with a shallow circulation with a maximum divergence below 700 hPa might form less mid-level cloud than a model with a shallow circulation and a peak divergence at 500 hPa.

If the overall strength of lower-tropospheric mixing is, in fact, regulated by bulk precipitation efficiency arguments such as those outlined above, then this raises the intriguing possibility that the lower-tropospheric mixing and the cloud feedback in a given model might be quite similar in magnitude in the standard and ConvOff configurations, even if much of the mixing is done by the convective parametrization in the standard configuration. A large-scale constraint on lower-tropospheric mixing could mean that resolved and parametrized turbulent mixing adjust to compensate for an absence of parametrized convective mixing in the ConvOff experiments. This question could be investigated in future work by directly quantifying the lower-tropospheric mixing associated with parametrized convection, turbulent and resolved mixing, for example by using temperature and humidity budget terms as in Zhang *et al.* [10] and Webb & Lock [7].

Our results also indicate that models with more low level clouds in the most stable areas of the tropics tend to have more positive low feedbacks in those regions. This finding is consistent with the expectations from the ‘beta feedback’ hypotheses of Brient & Bony [13]. Our results also hint that models with larger values of longwave CRE in the most stable regimes tend to have more positive cloud feedbacks across the tropics. Studies with LES have demonstrated that an enhanced free-tropospheric greenhouse effect can reduce turbulent mixing and cloudiness in the subtropical boundary layer [15]. There is also observational support for cirrus clouds breaking up low level cloud in the current climate [92]. In the subsidence regions, the subsidence rate is related to the lapse rate and the amount of radiative cooling [93]. In the absence of substantial differences in lapse rate, models with more upper level cloud or larger IWPs in the subsidence regions will have weaker radiative cooling in the upper troposphere, reducing the subsidence rate at upper levels and making the circulation more bottom heavy (i.e. having stronger subsidence at lower levels compared with upper levels). In a similar vein, models with more low level clouds will have more radiative cooling at low levels, which will enhance subsidence at low levels, also making the circulation more bottom heavy. Given that we find higher sensitivity models to have more low clouds and more cirrus in the more stable regimes in the tropics, this could explain why Sherwood *et al*. [21] found higher sensitivity models to have more shallow circulations. Although our analysis did not find any correlations between measures of the shallow circulation and cloud feedback which were robust across both of our ensembles, it remains possible that lower-tropospheric mixing associated with such resolved circulations contributes to inter-model spread in cloud feedback in our models, if not to a detectable degree.

We consider the potential mechanisms outlined above to be plausible but they are not the only possibilities. These and other hypothesized mechanisms could be tested in a number of ways in future process simplification experiments. For example, the precipitation efficiency in models could be reduced by modifying the cloud microphysics to alter the ease of raining from mid-level clouds over warm SSTs. Alternatively, cloud condensation at mid-levels could be suppressed by re-evaporating cloud water. If the ideas outlined above are correct, then this would be expected to force more condensation to occur at higher levels, increase vertical transports of water vapour and boundary layer drying, reduce MSE near the top of the boundary layer and strengthen positive low level cloud feedbacks. The idea that having more low-level cloud in stable regions of the tropics results in a more positive feedback could be further tested by tuning low-level cloud fractions, extending the approach of Brient & Bony [13] to a wider range of models. More specifically, the ‘beta feedback’ hypothesis of Brient & Bony [13] could be tested in more models by suppressing the longwave CREs of low level clouds, building on the approach of Fermepin & Bony [94]. Similarly, the longwave radiative impact of cirrus clouds on low-level cloud feedbacks could be tested by making ice clouds transparent to longwave radiation. Such experiments might be more straightforward or inexpensive to perform in more idealized model configurations; for example, the CFMIP aquaplanet configuration which is zonally symmetric has no seasonal cycle and has been shown to reproduce the inter-model spread in cloud feedbacks on more realistic configurations very effectively [5,75].

## 5. Summary and conclusions

In this study, we have demonstrated a new approach for investigating the processes contributing to inter-model spread in cloud feedback. We have investigated the sensitivity of cloud feedbacks to the use of convective parametrization by repeating the CMIP5/CFMIP-2 AMIP/AMIP +4K uniform sea surface temperature perturbation experiments with 10 climate models which have their convective parametrizations turned off. This is the first study to report results from a substantial ensemble of models without parametrized convection.

Integrations without parametrized convection were successfully performed without increasing model resolution, although other minor changes such as shorter timesteps were required in some cases to maintain model stability. Some aspects of present-day model performance were degraded in these ‘ConvOff’ experiments compared with the standard versions. The ConvOff versions generally have more negative values of the shortwave and net CRE across the tropics, associated with increased low-level fractions and LWPs. The ConvOff models also have a tendency for increased values of longwave CRE in low cloud regimes, owing to increases in high-level cloud fractions and/or IWP. Increased shortwave reflection from low-level clouds in particular results in increased biases in the top-of-atmosphere radiative balances of the ConvOff models.

The overall range in global cloud feedback in the standard model configurations is maintained in the ConvOff experiments, increasing by 22%. The models all show increases in low level cloud fraction when parametrized convection is switched off, substantially increasing the shortwave radiation reflected to space. Applying a simple bias correction method to allow for differences in present-day global mean net CRE substantially reduces the differences between the global mean cloud feedbacks with and without parametrized convection in the individual models. The cloud feedbacks in the two ensembles become strongly correlated, with the Convoff experiments exploring 85% of the overall range from the standard models. This correlation, and the fact that the models are capable of exploring much of the overall range in cloud feedbacks without convective parametrizations active, strongly suggests that although parametrized convection influences the strength of the cloud feedbacks substantially in some models, aspects of model formulation other than convective parametrization ultimately determine the overall range in the cloud feedbacks in the models examined here.

It is in principle possible that changes to the details of convective parametrizations which are not included in our current ensemble could have larger impacts on global cloud feedbacks than those seen here. However, our current standard and ConvOff ensembles already span the range in cloud feedbacks typically seen in climate models. Hence, adding new models or different convection schemes to our current ensembles would not affect our finding that the models can explore much of the range of contemporary cloud feedbacks without parametrized convection.

We have introduced a new approach for diagnosing cloud regimes over the low-latitude oceans. The ALPI is a hybrid index which combines the benefits of LTS for isolating stable low cloud regimes and surface precipitation rate for identifying strongly precipitating regimes of deep convection, making a continuous transition between them in LTS/precipitation space. ALPI composites of cloud feedbacks over the tropical oceans show that the largely positive shortwave cloud feedbacks in shallow cloud regimes in the models are still present in the ConvOff experiments, indicating that processes other than parametrized convection must be responsible. Our results also indicate that in the absence of convective parametrization the inter-model spread in net and shortwave cloud feedback is not greatly changed in stable regimes where stratocumulus clouds are expected to dominate the feedbacks. Meanwhile, it increases slightly in the regimes where we expect shallow trade cumulus clouds to dominate.

Convective parametrizations do however make a substantial contribution to inter-model spread in the longwave cloud feedbacks in strongly precipitating regions of the tropics; the spread of this feedback is substantially reduced in the ConvOff experiments. This local effect is however clearly not large enough to have a substantial effect on the overall range of the global net cloud feedback.

We have also assessed the impact of convective parametrizations on the present-day simulation of various cloud variables and looked for relationships between them and the cloud feedbacks. We have identified a number of statistically significant relationships between present-day cloud properties within ALPI regimes and the net cloud feedbacks within those regimes and also those averaged over the entire low-latitude ocean area, which are robust across models with and without parametrized convection. Models with more low cloud and stronger values of the shortwave CRE in the most stable regimes in the tropics tend to have more positive cloud feedbacks within that regime, consistent with the findings of Brient & Bony [13], who found that subtropical feedbacks in parameter-perturbed versions of the single column version of IPSL-CM5A-LR were stronger in cases where more low level cloud and stronger values of shortwave CRE were present in the control case. Additionally, models with larger values of the longwave CRE (and more high level cloud or larger IWPs) in the most stable areas of the tropics tend to have stronger cloud feedbacks averaged across the low-latitude oceans. We also found that models with the least mid-level cloud in the deep convection and trade cumulus regimes tend to have the most positive feedbacks both within the trade cumulus regimes and averaged over the low-latitude oceans. Additionally, models with less MSE near the top of the boundary layer in the trade cumulus regimes tend to have more positive cloud feedbacks there.

We have discussed a number of possible physical mechanisms which could explain our results, and how these and other ideas could be tested in the future by performing further process simplification experiments. If a robust interpretation of such results can be confirmed by such sensitivity experiments in the future, then the relationships that we have identified between feedbacks and present-day cloud variables could form the basis for a new set of emergent constraints on tropical cloud feedback. Although mid-level clouds in strongly precipitating regions are somewhat difficult to observe, the inclusion of cloud simulators in a wider range of models based on active instruments such as CloudSat and CALIPSO (e.g. [6]) would support a quantitative evaluation of this aspect of model performance.

More generally speaking, the roles of processes other than parametrized convection in contributing to inter-model spread in cloud feedback could be explored further by modifying other aspects of model physics, either by switching them off as we have done here, or by replacing particular schemes with the same simplified version in different models. The present-day simulation of shallow clouds is known to be highly sensitive to the details of turbulent mixing and entrainment parametrizations in models. A recent LES study by Bretherton & Blossey [17] demonstrated a positive cloud feedback associated with an entrainment liquid-flux mechanism, where an increased cloud layer humidity flux in a warmer climate induces an entrainment liquid-flux adjustment that dries the stratocumulus cloud layer. Turbulent entrainment parametrizations could be switched off to assess their contributions to inter-model spread in cloud feedback. Alternatively, the turbulent mixing schemes in the models could be replaced with a simple and consistent alternative, for example one based on a Richardson number-dependent vertical diffusivity term which would confine mixing within the boundary layer (as opposed to a constant diffusivity which would have undesirable effects near the tropopause). Additionally, the role of the large-scale circulation in contributing to inter-model spread in cloud feedback could be explored. For example, the importance of the large-scale component of the lower-tropospheric mixing mechanism proposed by Sherwood *et al*. [21] (which is argued to vary according to the depth of the subtropical circulation) could be explored by applying artificial diabatic heating terms to the models designed to change the depth of the circulation. As pointed out above, this might be more straightforward to do in aquaplanet configurations. We plan to develop the SPOOKIE approach further in the future by designing sensitivity tests for GCMs which target such questions directly.

We hope that the data used in this study will be useful to investigate the impact of convective parametrizations on many other aspects of climate model simulations. For example, we plan to write a follow-up paper that assesses the impact of parametrized convection on various aspects of present-day climate. We also plan to make the data from the ConvOff simulations available to the wider scientific community in the near future. For more details, please contact the corresponding author or refer to the CFMIP website (http://www.cfmip.net).

## Data accessibility

The CMIP5 climate model output data used in this study are available via the Earth System Grid Federation portal—see http://cmip-pcmdi.llnl.gov/cmip5/data_getting_started.html. Data from other experiments performed specifically for this study are owned by the relevant modelling groups but are available on request by contacting the corresponding author.
